# Changes in Pregabalin Dispensing to Australians with Workers’ Compensation Claims for Low Back Pain Following the Listing of Pregabalin on the Pharmaceutical Benefits Scheme

**DOI:** 10.1007/s10926-025-10276-5

**Published:** 2025-02-12

**Authors:** Michael F. Di Donato, Christina Abdel Shaheed, Alex Collie, Christopher G. Maher, Stephanie Mathieson

**Affiliations:** 1 Healthy Working Lives Research Group, School of Public Health and Preventive Medicine, Monash University, 553 St Kilda Road, Melbourne, Victoria 3004 Australia; 2 Institute for Musculoskeletal Health, Sydney Local Health District, Sydney, NSW Australia; 3 Sydney Musculoskeletal Health, Faculty of Medicine and Health, The University of Sydney, Sydney, NSW Australia; 4 Sydney School of Public Health, Faculty of Medicine and Health, The University of Sydney, Sydney, NSW Australia; 5 Australian and New Zealand Centre of Research Excellence in Low Back Pain (ANZBACK), Sydney, NSW Australia

**Keywords:** Workers' compensation, Pain medicine, Low back pain

## Abstract

**Objectives:**

We sought to identify whether the inclusion of pregabalin on the list of medicines subsidised by the Australian government in 2013 resulted in changes to the dispensing of pregabalin to Australians with workers’ compensation claims for low back pain.

**Methods:**

Using a sample of workers’ compensation claims and payments data (2010–2018), we measured the prevalence of pregabalin dispensing and time to first pregabalin dispensing in workers whose claim began before, during or after pregabalin was listed on the Pharmaceutical Benefits Scheme (PBS) with binary logistic and Cox proportional hazards models. We used interrupted time-series analyses to measure changes in the monthly number, percentage of pain medicines, percentage of gabapentinoid dispensings, and median cost per dispensing of pregabalin.

**Results:**

Of the 17,689 workers included in the study, 13.7% (n = 2431) were dispensed pregabalin during the study period. Workers in the groups whose claim occurred before or during when pregabalin was listed on the PBS were at significantly lower odds of being dispensed pregabalin than the group whose claim began after PBS listing (OR 0.20, 99% CI 0.15, 0.25 and OR 0.40, 99% CI 0.33, 0.48, respectively). There were significant step increases in the number of pregabalin dispensings (26.3%, 99% CI 6.2%, 50.3%), percentage of pain medicines that were pregabalin (29.3%, 99% CI 1.5%, 64.9%), and percentage of gabapentinoid dispensings that were pregabalin (13.9%, 99% CI 7.5%, 20.6%). There was a significant step decrease in the median cost per pregabalin dispensing (− 61.8%, 99% CI − 66.8%, − 56.1%).

**Conclusion:**

Listing pregabalin on the PBS saw significant increases in the prevalence, number and percentage of pain medicines, and significant decreases in time to first pregabalin dispensing and cost.

**Supplementary Information:**

The online version contains supplementary material available at 10.1007/s10926-025-10276-5.

## Introduction

Pregabalin has increasingly been used outside approved use, or “off-label”, for low back pain [1–3]. However, literature continues to highlight that gabapentinoids (i.e. pregabalin and gabapentin) are not effective in relieving symptoms of conditions that are often associated with off-label use, such as low back pain and sciatica, and are frequently associated with harmful side effects [2, 4]. Pregabalin, indicated for use to manage neuropathic pain conditions, such as post-herpetic pain, was originally added to the list of medicines subsidised by the Australian government (i.e. the Pharmaceutical Benefits Scheme (PBS)) on 1st March 2013 [5]. While available prior to this date through private insurance or out-of-pocket purchase, subsidisation through the PBS meant prices were markedly reduced: the out-of-pocket expense per dispensing (i.e. box) decreased from over 100 to 36 Australian dollars. Analysis of PBS data by the Department of Health Drug Utilisation Sub-Committee highlighted that over 290,000 people were treated with PBS-subsidised pregabalin in the first year of listing [5].

Workers in Australia with a work-related injury are eligible to receive income and health care support (including medicines) from a workers’ compensation scheme [6]. Where the prescribed medicine is an eligible PBS medicine, the workers’ compensation scheme will fund the out-of-pocket expense [7, 8]. If the medicine is a private prescription, the scheme will also fund the medicine. Either way, an injured worker will not incur an expense for medicines prescribed for their injury or illness. Similar to other countries with workers’ compensation schemes, low back pain is a common reason for a workers’ compensation claim in Australia [9]. Recent publications have highlighted that the rate of gabapentinoids dispensed to those with workers’ compensation claims in North America has increased over the past decade [10, 11]. Evidence in Australia has highlighted increasing use of pregabalin in the general population since its inclusion on the PBS [1, 12]. Finally, literature has also pointed to the varying use of other pain medicines that may not be effective for low back pain, such as opioid analgesics, antidepressants and benzodiazepines, that may be used in conjunction with pregabalin [11, 13–16].

In the context of such evidence, it is prudent to investigate whether listing pregabalin on the PBS and reducing its price resulted in any changes to use in the Australian workers’ compensation sector. Prior to PBS listing, the workers’ compensation scheme funded the full private prescription cost of pregabalin, and after PBS listing the scheme funded the full out-of-pocket subsidised cost. We therefore hypothesised that since there were no appreciable changes to expenses for the worker following PBS listing, there should not have been any changes to pregabalin use. We sought to identify whether listing pregabalin on the PBS resulted in changes to the prevalence, frequency and cost of pregabalin dispensed to Australians with workers’ compensation time loss claims for low back pain.

## Methods

### Setting

We conducted this study in the Australian state of Victoria. Approximately, 90% of the Victorian labour force (2.85 million people at the time of pregabalin’s listing on the PBS) are covered for injuries and illness incurred in the course of employment by the state’s workers’ compensation scheme [17, 18]. In most cases, employers fund the first 10 business days income replacement and approximately $700AUD medical expenses. Once these thresholds have been passed, the workers’ compensation scheme funds income replacement and reasonable and necessary health care. Claims for prescription medicines are lodged by pharmacists with one of the insurance agents who manage the workers’ compensation scheme [19]. Where a medicine is listed on the PBS and the worker is eligible for the PBS (i.e. they are registered with the Medicare Benefits Scheme), then the scheme funds the out-of-pocket expense for the worker.

### Data Source

This study uses a sample of workers’ compensation time loss claims from the Victorian workers’ compensation scheme provided by the regulator, WorkSafe Victoria [20]. The sample contained claims (e.g. age, sex, occupation, claim dates, one record per claim) and medicines data (e.g. type of medicine, strength, date dispensed, quantity dispensed, multiple records per claim). Claims and medicines data were cleaned in a similar manner, checking for illogical values and missing data. Medicine dispensed was assigned to the appropriate Anatomical Therapeutic Chemical (ATC) code [21].

### Sample

We included workers with accepted workers’ compensation claims for low back pain received by the insurer between 01 January 2010 and 31 December 2017. Only workers with at least one day of income replaced by the workers’ compensation scheme were included. That is, claims where the worker had exceeded employer excess and was absent from work for at least two weeks. Low back pain was defined using the type of occurrence classification system (see supplementary materials) [22]. Eligible workers were aged between 15 and 80 years.

We included pain medicines dispensed to eligible workers 31 days before to 730 days (i.e. two years) after the date the insurer received the workers’ compensation claim (i.e. the latest medicine dispensed before 31 December 2019). Pain medicines were identified using ATC level 2 codes and included medicines for the musculoskeletal and nervous system (see supplementary materials). Specific categories of pain medicines that have more commonly been used for low back pain were identified to be used as covariates in analyses. These included gabapentin (ATC level 5 code N02BF01), opioids (ATC level 3 code N02A), antidepressants (ATC level 3 code N06A) and benzodiazepines (ATC level 4 codes N03AE, N05BA and N05CD). These medicines were also selected as they were prescription medicines, and more accurately recorded in the data than over-the-counter medicines (e.g. paracetamol or non-steroidal anti-inflammatories). Pregabalin was identified by the ATC level 5 codes N02BF02. We included medicines 31 days prior to the insurer received date because, while infrequent, health care may be retrospectively reimbursed. The follow-up period of two years is similar to previous studies of medicines in workers’ compensation, allowing adequate comparison.

### Outcome Variables

Primary outcomes were the number and percentage of workers dispensed pregabalin in the two years since an insurer received their claim, the median days to first pregabalin dispensing and the percentage of workers dispensed gabapentin and three other categories of pain medicines (i.e. opioids, antidepressants and benzodiazepines) before and after the first pregabalin dispensing. Secondary outcomes included the monthly number of pregabalin dispensings, monthly percentage of pain medicine dispensings that were pregabalin, monthly percentage of gabapentinoid dispensings that were pregabalin and the monthly median cost per pregabalin dispensing in Australian dollars.

### Covariates

We included a number of covariates in analyses. Worker sex was reported in binary terms and age group in 10-year bands (i.e. 15–24, 25–34, 35–44, 45–54, 55–64 65 + years). Worker occupation was reported in Australian Standard Classification of Occupations major groups [23]. Employment type was collapsed to full-time, part-time, casual or other, and employer size was retained as the original government, large, medium and small. Socioeconomic status was identified by the Index of Relative Socioeconomic Advantage and Disadvantage quintile for a worker’s residential postcode [24], with the middle three quintiles collapsed into one level for explanatory parsimony. Worker remoteness was defined by the Accessibility and Remoteness Index of Australia [25]. Finally, binary indicators were created that flagged whether a worker had been dispensed gabapentin (ATC level 5 code N02BF01), opioids (ATC level 3 code N02A), antidepressants (ATC level 3 code N06A) or benzodiazepines (ATC level 4 codes N03AE, N05BA and N05CD) during the same two-year period.

### Analysis

**Fig. 1:**
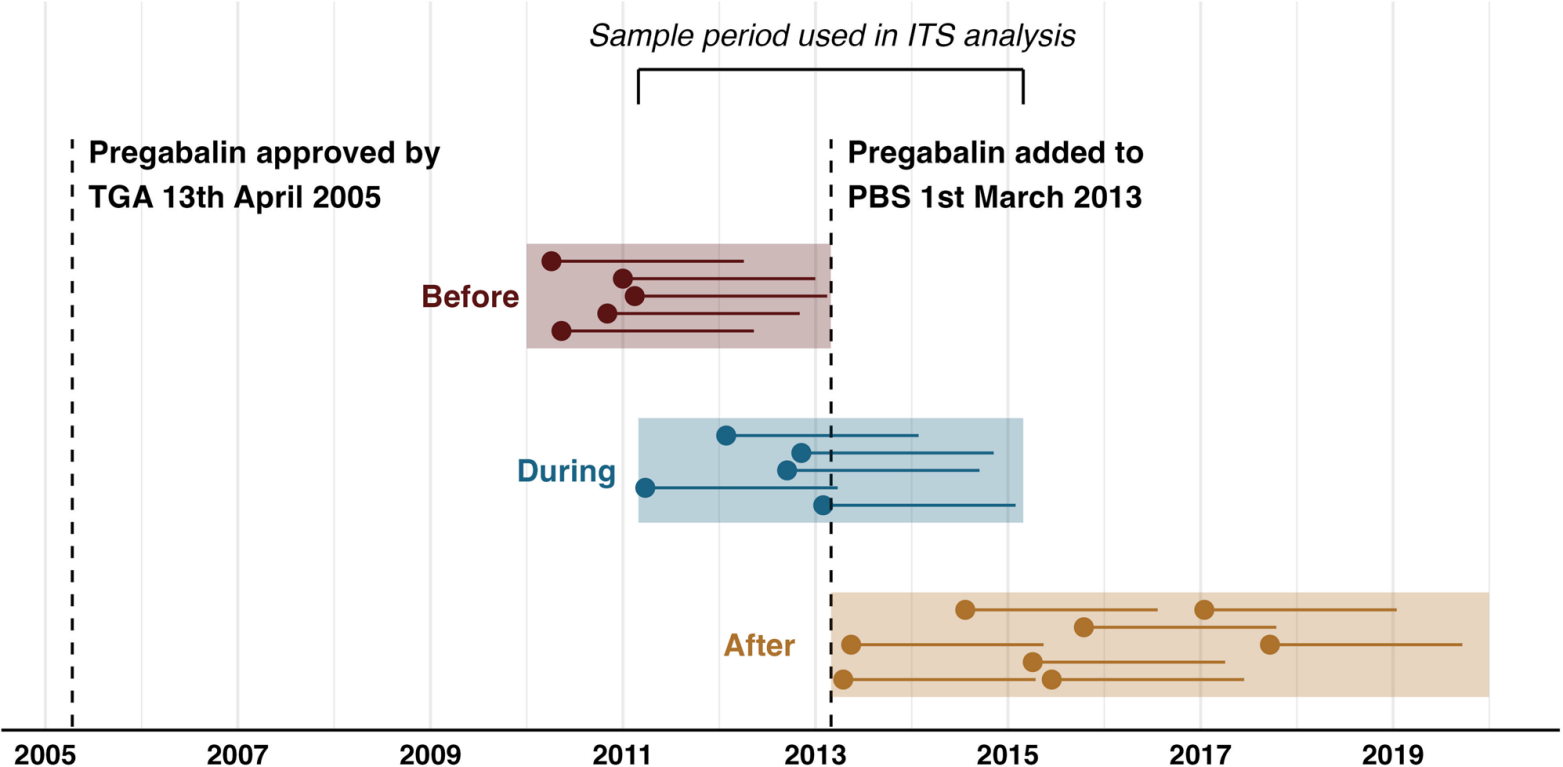
Timeline to pregabalin listing on PBS overlaid with study sample. Points represent example dates insurer received a claim, lines represent two-year (i.e. 730-day) follow-up period. Before, during and after PBS listing groups for logistic regression and four-year sample for ITS analysis are highlighted

We grouped workers by whether the first two years since the insurer received their claim occurred before, during or after pregabalin was listed on the PBS. That is, a worker would be assigned to the “before” pregabalin PBS listing group if their claim was received by the insurer prior to 01 March 2011, “during” if the claim was received between 01 March 2011 and 01 March 2013 and “after” if the claim was received after 01 March 2013. This grouping and the timeline of pregabalins listing on the PBS are displayed in Fig. 1.

We used descriptive statistics to report the number and percentage of workers who were dispensed pregabalin at any time in the first two years of their claim, and the median and inter-quartile range (IQR) days from the date the insurer received the claim to the first pregabalin dispensing in each group. We used binary logistic regression to compare the likelihood of being dispensed pregabalin between groups, and a Cox proportional hazards model to compare the time to first pregabalin dispensing between groups. Both models adjusted for worker sex, age group, employment type, employer size, occupation, socioeconomic status, remoteness and being dispensed gabapentin, opioids, antidepressants or benzodiazepines in the same follow-up period. The “after” group was used as the reference group due to size. Workers with missing data were excluded from both models. We reported results as odds ratios (logistic regression) and hazards ratios (Cox regression) with 99% Confidence Intervals (CI). Results were considered significant if *p* < 0.01.

We then used descriptive statistics to identify the percentage of workers dispensed pregabalin who were (1) not dispensed another category of pain medicine, (2) dispensed pregabalin before ever being dispensed another pain medicine, or (3) were dispensed pregabalin after ever being dispensed another pain medicine. We generated these statistics for each of gabapentin, opioids, antidepressants and benzodiazepines.

We produced a time series with monthly intervals of the number of pregabalin dispensings, percentage of pain medicine dispensings that were pregabalin, percentage of gabapentinoids that were pregabalin and the median cost per pregabalin dispensing for two years before to two years after pregabalin was listed on the PBS (i.e. March 2011 to March 2015) (see Fig. 1). This time-series subsample meant that not all dispensings or workers from our sample were included but accounted for potential low numbers of dispensings as the number of workers increased from the sample start date, which may have affected count models. We used descriptive statistics to report the mean and standard deviation (SD) of monthly outcomes in the two years before and two years after PBS listing. A negative binomial model was used to statistically measure changes in the number of pregabalin dispensings. Generalised least squares models were used to measure changes in the percentage of pain medicine dispensings that were pregabalin, percentage of gabapentinoids that were pregabalin and median cost per pregabalin dispense. We tested for seasonality by adding six sine and six cosine terms to initial models. Seasonality terms that were significant (*p* < 0.05) were retained in final models. Autoregressive moving average methods were used to assess models for autocorrelation and partial autocorrelation. Akaike and Bayesian Information Criteria were used to compare and select final models [26, 27]. We reported results in percentage step (i.e. immediate change) and trend (i.e. gradual change) change with 99% CIs by log-transforming results of generalised least squares models and taking the percent change in incidence rate ratio of the negative binomial model. Results were statistically significant where *p* < 0.01.

We performed analyses in RStudio using R 4.2.2 and a number of R packages (see supplementary materials). The Monash University Human Research Ethics Committee (MUHREC) approved this study (Project ID 30718).

## Results

A total of 17,689 workers had a workers’ compensation time loss claim for low back pain in the study period. 13.7% (*n* = 2431) of these workers were dispensed pregabalin in the two years since the insurer received their claim. There were 17,520 pregabalin dispensings (91.0% of gabapentinoids) over the study period accounting for 9.8% of pain medicine dispensings. Those workers dispensed pregabalin received a median of 4 (IQR 1, 10) dispensings.

### Prevalence and Timing of Pregabalin Dispensings

Descriptive statistics revealed the greatest number (*n* = 1761) and percentage (17.0%) of workers dispensed pregabalin occurred in those whose first two years of claim were after PBS listing (i.e. insurer received claim after 01 March 2013) (see Table 1). Workers whose first two years of claim occurred before (i.e. insurer received claim prior to March 2011) or during (i.e. insurer received claim between March 2011 and March 2013) PBS listing were at significantly lower odds of being dispensed pregabalin than those whose claim began after PBS listing (OR 0.20, 99% CI 0.15, 0.25 and OR 0.40, 99% CI 0.33, 0.48, respectively). The 179 workers whose first two years of claim occurred prior to PBS listing were not dispensed pregabalin until a median of 284 days (IQR 114.5, 456.5) from when the insurer received the claim. A similar time to first dispensing was observed in workers with claims during the PBS listing, with both groups having a significantly longer duration to first dispensing than workers whose claims began after PBS listing (Before: HR 0.56, 99%CI 0.45, 0.68 and During: HR 0.55, 99% CI 0.48, 0.64).

**Table 1:** Proportion of workers dispensed pregabalin in the first two years of claim and the time to first pregabalin dispense

Insurer received a worker’s claim	Total workers	Workers ever dispensed pregabalin			Time to first pregabalin dispense		
	*N*	*N* (%)	OR (99%CI)^a^	*p*	Median (IQR) Days	HR (99%CI)^b^	*p*
Before PBS listing (< March 2011)	2692	179 (6.6)	0.20 (0.15, 0.25)	< 0.001	284.0 (114.5, 456.5)	0.56 (0.45, 0.68)	< 0.001
During PBS listing (March 2011 to March 2013)	4658	491 (10.5)	0.40 (0.33, 0.48)	< 0.001	278.0 (126.0, 449.0)	0.55 (0.48, 0.64)	< 0.001
After PBS listing (> March 2013)	10,339	1761 (17.0)	1.00 (ref)	–	84.0 (21.0, 238.0)	1.00 (ref)	–
All workers (January 2010 to December 2017)	17,689	2431 (13.7)	–	–	124.0 (34.0, 318.5)	–	–

^a^Binary logistic regression model, Odds Ratio (OR) (99% Confidence Interval)

^b^Cox proportional hazards model, Hazards Ratio (HR) (99% Confidence Interval)

^c^Both models adjusted for worker sex, age, employment type, employer size, occupation, socioeconomic status, remoteness, whether a worker was dispensed opioids, antidepressants or benzodiazepines in the same period (see supplementary material for full models)

### Pregabalin Dispensed Relative to Other Pain Medicines

**Fig. 2:**
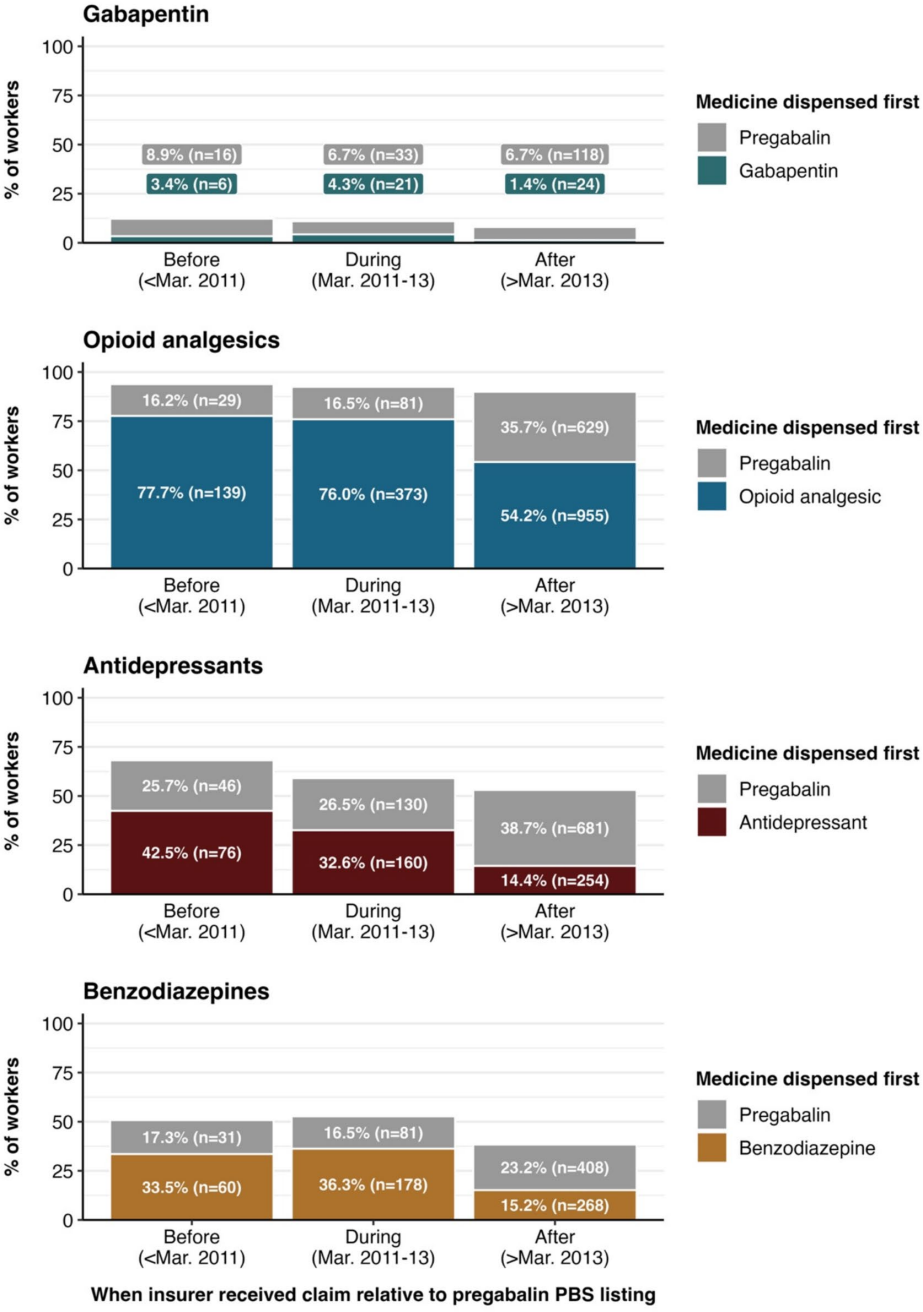
Percentage of workers dispensed pregabalin (*n* = 2431) who were dispensed gabapentin, opioids, antidepressants and benzodiazepines relative to pregabalin, stratified by whether the insurer received a worker’s claim before (< March 2011), during (March 2011 to March 2013) or after (> March 2013) pregabalin was listed on the PBS

Nearly all workers in each group who were dispensed pregabalin were also dispensed opioids, while very few were also dispensed gabapentin (see Fig. 2). While only 16.2% and 16.5% of workers with claims before and during PBS listing were dispensed pregabalin prior to opioids, this increased to 35.7% of workers with claims after PBS listing. The percentage of workers dispensed pregabalin prior to antidepressants and benzodiazepines was similarly highest in workers with claims after pregabalin was listed on the PBS.

### Comparisons of Pregabalin Trends

**Fig. 3:**
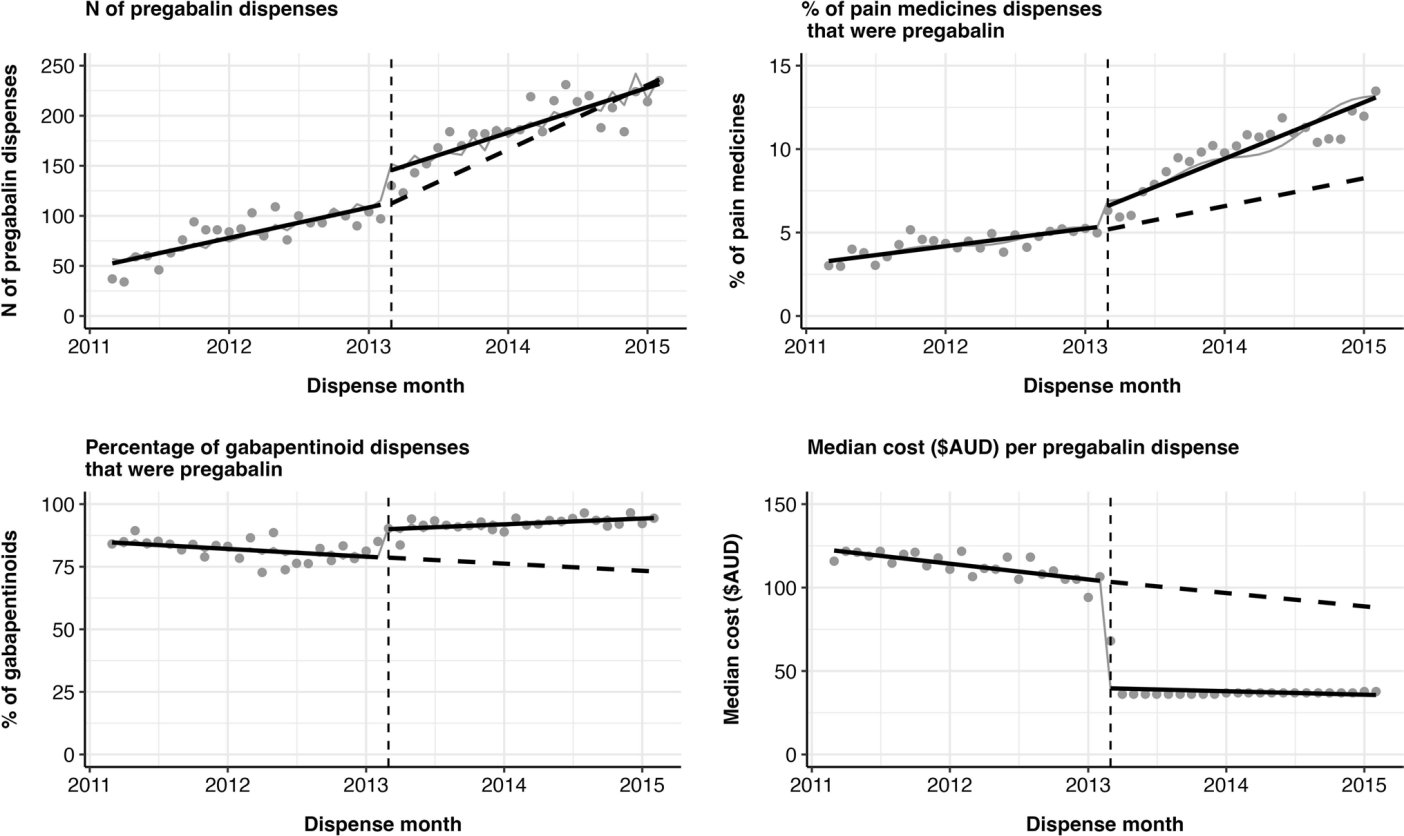
Interrupted time-series analyses of step and trend changes in pregabalin dispensing before and after PBS listing of pregabalin. Points represent original data, solid lines represent modelled trends and dotted lines represent modelled counterfactual trends

There was a significant step increase of 26.3% (99% CI 6.2%, 50.3%) in the number of pregabalin dispensings from March 2013 following PBS listing (see Fig. 3 and Table 2). There was also a significant step increase of 29.3% (99% CI 1.5%, 64.9%) in the percentage of pain medicines that were pregabalin. The majority of gabapentinoid dispensings were pregabalin, but there was a significant 13.9% (99% CI 7.5%, 20.6%) step increase and 0.5% (99% CI 0.1 to 0.9%) trend increase in the percentage of gabapentinoid dispensings that were pregabalin after PBS listing. Finally, there was a significant step decrease of − 61.8% (99% CI − 66.8% to − 56.1%) in the median dispensing cost of pregabalin.

**Table 2:** Interrupted time-series analyses of step and trend changes in pregabalin dispensing before and after PBS listing of pregabalin

	2 years pre PBS listing	2 years post PBS listing	Step change^a^	*p*	Trend change^b^	*p*
			% (99% CI)^c^		% (99% CI)	
Mean (SD) monthly N of pregabalin dispensed	81.7 (21.4)	188.5 (30.6)	26.3% (6.2%, 50.3%)	< 0.001	− 1.1% (− 2.4%, 0.2%)	0.024
Mean (SD) monthly % of pain medicines dispensings that were pregabalin	4.3 (0.7)	9.9 (2.0)	29.3% (1.5%, 64.9%)	0.009	0.8% (− 1.3%, 3.0%)	0.312
Mean (SD) monthly % of gabapentinoids that were pregabalin	81.8 (4.4)	92.2 (2.6)	13.9% (7.5%, 20.6%)	< 0.001	0.5% (0.1%, 0.9%)	0.002
Median (IQR) cost per pregabalin dispensing ($AUD)	113.9 (75.0, 128.0)	36.9 (31.8, 36.9)	− 61.8% (− 66.8%, − 56.1%)	< 0.001	0.2% (− 0.8%, 1.3%)	0.551

^a^Step change immediately following listing of pregabalin on PBS

^b^Trend change in two years following listing of pregabalin on PBS

^c^Percentage change with 99% confidence intervals (99% CI)

## Discussion

Subsidising the cost of pregabalin resulted in increased workers’ compensation scheme funded dispensing to workers in Australia with low back both in the total number of pregabalin dispensings and as the proportion of all medicines dispensed for pain management. The proportion of workers with a low back claim who were dispensed pregabalin in the first two years of their claim nearly tripled since pregabalin was listed on the PBS in 2013, and the median time to first dispensing significantly decreased by approximately 70% to 84 days. There were significant step increases in the number and percentage of pregabalin dispensings following PBS listing, although both metrics were already trending upwards. Pregabalin was the predominant gabapentinoid dispensed to workers in our sample, but there was a significant step increase in the ratio to gabapentin. Unsurprisingly, the median cost per pregabalin dispensing dramatically reduced to the out-of-pocket PBS co-payment price.

Listing pregabalin on the PBS meant it was subsidised at a reduced price and accessible to more patients than only being available for private purchase since its approval date as early as 2005 [28]. The PBS is administered by the Australian Government and funded by general taxation, whereas workers’ compensation schemes are state-based and funded by employers [29, 30]. Our findings indicate that the Victorian workers’ compensation scheme shifted from funding the full private price of pregabalin to the PBS co-payment amount (i.e. AUD36.90 in 2013). The substantial reduction in cost per prescription for the workers’ compensation scheme may have reduced claim manager concerns about approving more expensive and infrequently used medicines and may be one reason for the rise in pregabalin prescriptions in our sample. That is, the difference in cost moved from the insurer to the taxpayer. However, it is unlikely that reduced costs were the only reason contributing to increases in dispensings.

Substantial increases in prescribing of pregabalin to the general population for approved conditions or off-label use have been observed since PBS listing [1]. It is likely that some of the change observed in workers’ compensation is due to the overall upward trend. PBS listing may have increased awareness and confidence to prescribe among physicians, particularly in the context of growing pressure against opioid analgesics. There was also substantial professional education of pain conditions conducted by the manufacturer of pregabalin following the 2012 decision of the Pharmaceutical Benefits Advisory Committee to approve pregabalin for PBS listing [31, 32]. Given General Practitioners (a primary point of care for workers’ compensation in Australia) tend to choose pain medicines for low back pain based on prior experience rather than guidelines [33], education by manufacturers may have had a significant influence on the increasing prescribing of pregabalin.

Our findings of increasing use of pregabalin align with studies in the North American workers’ compensation setting [10, 11, 34]. However, there were stark differences in the type of gabapentinoid, with gabapentin, rather than pregabalin, the predominant option for compensated American workers. Liu et al. [11] proposed that higher rates of gabapentin use may be due to restrictions on pregabalin implemented by workers’ compensation schemes due to rising costs. However, it is also important to note that our study specifically examined workers with claims for low back pain, whereas literature from North American studies examined claims for any condition.

There is increasing awareness of the limited effectiveness of pregabalin for low back pain, and harmful side effects associated with use [2]. More severe consequences, such as overuse requiring ambulance attendance, are increasing [35]. These potential hazards are acknowledged in the public health setting by the inclusion of pregabalin on local prescription-drug monitoring programmes, such as SafeScript in the state of this study (Victoria), SafeScript NSW in New South Wales, ScriptCheckSA in South Australia, QScript in Queensland [36–39]. However, given that prescribing for low back pain is considered “off-label”, the prevalence observed in our study is still relatively high. At a broader level, our findings indicate that while health care funding sources are technically separate, changes to one have the capacity to cause changes in another. Prior research has demonstrated how workers move between systems, and that major policy changes in one system can lead to unintended consequences for another [40, 41]. Workers’ compensation scheme regulators and insurers should be vigilant of major changes to neighbouring health care and social systems.

### Strengths, Limitations and Future Research

Our study benefited from a large sample of highly detailed data, enabling us to examine thousands of pregabalin dispensings over a long study period. We utilised robust statistical techniques to measure both the changes in pregabalin prevalence and time to use in workers and changes in pregabalin dispensing trends. Our study is not without limitations. The data used only capture medicines funded by workers’ compensation and it is possible that workers may have obtained pregabalin funded by the PBS, private health insurance or out-of-pocket expenses. We acknowledge there were relatively few workers dispensed pregabalin, particularly those whose first two years of claim were prior to PBS listing. This smaller number of workers may have reduced the precision of our estimates. Finally, workers included in our study represent those with potentially more serious pain and disability; they had to have been away from work for two weeks to be eligible for inclusion. This may reduce the generalisability to other populations. In light of these limitations, future research could first aim to link workers’ compensation data to PBS or private health insurance data, enabling a complete picture of medicines use. Furthermore, research could be expanded to additional Australian states and territories. While workers’ compensation schemes have the same objectives in Australia, there are critical differences in policies and practices that may affect outcomes. For example, unlike most other Australian states, Victoria has an AUD700 medical excess, meaning the first AUD700 of medical expenses are not recorded in our data. Lastly, future research could utilise more recent data to explore recent trends in pregabalin use.

## Conclusion

Adding pregabalin to the national pharmaceutical benefit scheme increased the number of dispensings, percentage of pain medicine dispensings and percentage of gabapentinoids dispensed to Australian workers with workers’ compensation claims for low back pain. Subsidising pregabalin through a taxpayer funded national scheme meant the price paid by the state-based workers’ compensation scheme decreased substantially to the PBS out-of-pocket price. These changes may have been driven by economic factors and marketing to physicians. Workers’ compensation schemes should remain vigilant of changes to other systems and schemes.

## Supplementary Information

Below is the link to the electronic supplementary material.

Supplementary file1 (PDF 129 KB)

## Data Availability

Data used in this paper are not available for distribution by the authors. The R analysis code used in analyses is available upon request.
