# Supplementary material for: Changes in Pregabalin Dispensing to Australians with Workers’ Compensation Claims for Low Back Pain Following the Listing of Pregabalin on the Pharmaceutical Benefits Scheme
Source: J Occup Rehabil. 2025 Feb 12;36(2):416–25. doi: 10.1007/s10926-025-10276-5 (PMC13099708; doi:10.1007/s10926-025-10276-5)
Supplement: Supplementary file 1 — Supplementary file1 (PDF 129 KB) [file 10926_2025_10276_MOESM1_ESM.pdf]

Changes in pregabalin dispensing to Australians with workers' compensation claims for low back pain following the listing of pregabalin on the pharmaceutical benefits scheme

Michael Di Donato[1,5], Christina Abdel-Shaheed[2,3,4], Alex Collie[1], Christopher G Maher[2,3] & Stephanie Mathieson[3]

1. School of Public Health and Preventive Medicine, Monash University
2. Institute for Musculoskeletal Health, Sydney Local Health District, Sydney, NSW Australia
3. Sydney Musculoskeletal Health, Faculty of Medicine and Health, The University of Sydney
4. Sydney School of Public Health, Faculty of Medicine and Health, The University of Sydney
5. Australian and New Zealand Centre of Research Excellence in Low Back Pain (ANZBACK)

Corresponding Author:

Dr Michael Di Donato

Healthy Working Lives Research Group, School of Public Health and Preventive Medicine, Monash University

553 St Kilda Road, Melbourne, Victoria 3004, Australia

michael.didonato@monash.edu, +61 9905 6417

Supplementary Table 1. Low back pain sample criteria

TOOCS Parameter	***Code***	***Description***
Nature of Injury	228	Trauma to muscles and tendons, not elsewhere classified
	229	Trauma to muscles and tendons, unspecified
	239	Soft tissue injures due to trauma or unknown mechanisms
	422	Disc displacement, prolapse, degeneration or hernia
	459	Back pain, lumbago and sciatica
	488	Spinal vertebrae and intervertebral discs diseases, not elsewhere classified
	489	Spinal vertebrae and intervertebral disc diseases, unspecified
	533	Muscle / tendon strain (non-traumatic)
Bodily Location of Injury	311	Lower back
Mechanism of Injury	Any	-
Agency of Injury	Any	-

Supplementary Table 2. Pain medicines included in analyses

ATC Code	***ATC Description***
M01	Antiinflammatory and antirheumatic products
M02	Topical products for joint and muscular pain
M03	Muscle relaxants
N01	Anesthetics
N02	Analgesics
N03	Antiepileptics
N05	Psychopleptics
N06	Psychoanaleptics

Supplementary Table 3. R packages used in analyses

Package	***Version***	***Reference***
MASS	7.3.58.3	https://cran.r-project.org/package=MASS
base	4.2.2	https://cran.r-project.org/package=base
broom	1.0.3	https://cran.r-project.org/package=broom
broom.mixed	0.2.9.4	https://cran.r-project.org/package=broom.mixed
datasets	4.2.2	https://cran.r-project.org/package=datasets
dplyr	1.1.3	https://cran.r-project.org/package=dplyr
forcats	1.0.0	https://cran.r-project.org/package=forcats
ggplot2	3.5.0	https://cran.r-project.org/package=ggplot2
ggpubr	0.6.0	https://cran.r-project.org/package=ggpubr
grDevices	4.2.2	https://cran.r-project.org/package=grDevices
graphics	4.2.2	https://cran.r-project.org/package=graphics
haven	2.5.1	https://cran.r-project.org/package=haven
lubridate	1.9.1	https://cran.r-project.org/package=lubridate
methods	4.2.2	https://cran.r-project.org/package=methods
naniar	1.0.0	https://cran.r-project.org/package=naniar
nlme	3.1.160	https://cran.r-project.org/package=nlme
patchwork	1.2.0	https://cran.r-project.org/package=patchwork
purrr	1.0.2	https://cran.r-project.org/package=purrr
readr	2.1.3	https://cran.r-project.org/package=readr
sandwich	3.0.2	https://cran.r-project.org/package=sandwich
scales	1.3.0	https://cran.r-project.org/package=scales
stats	4.2.2	https://cran.r-project.org/package=stats
stringr	1.5.1	https://cran.r-project.org/package=stringr
survival	3.4.0	https://cran.r-project.org/package=survival
survminer	0.4.9	https://cran.r-project.org/package=survminer
tibble	3.2.1	https://cran.r-project.org/package=tibble
tidyr	1.3.0	https://cran.r-project.org/package=tidyr
tidyverse	1.3.2	https://cran.r-project.org/package=tidyverse
utils	4.2.2	https://cran.r-project.org/package=utils

Supplementary Table 4. Proportion of workers dispensed pregabalin and the time to first pregabalin dispensing

	**Total Workers**	***Workers ever dispensed pregabalin***			***Time to first pregabalin dispense***		
		N (%)	*OR (99%CI)*	*p*	*Median (IQR) days*	*HR (99%CI)*	*p*
Insurer received claim				-			
Before PBS listing (< March 2011)	2,692	179 (6.6)	0.20 (0.15, 0.25)*	p<0.001	284.0 (114.5, 456.5)	0.56 (0.45, 0.68)*	p<0.001
During PBS listing (March 2011 to March 2013)	4,641	491 (10.6)	0.40 (0.33, 0.48)*	p<0.001	278.0 (126.0, 449.0)	0.55 (0.48, 0.64)*	p<0.001
After PBS listing (> March 2013)	10,356	1,761 (17.0)	1.00 (ref)	-	84.0 (21.0, 238.0)	1.00 (ref)	-
Sex				-			
Female	6,301	807 (12.8)	0.87 (0.71, 1.06)	0.062	125.0 (37.0, 311.0)	1.07 (0.92, 1.24)	0.263
Male	11,388	1,624 (14.3)	1.00 (ref)	-	123.0 (33.0, 322.2)	1.00 (ref)	-
Age group				-			
15-24 years	1,514	98 (6.5)	0.65 (0.45, 0.93)*	0.002	104.0 (29.2, 310.0)	0.99 (0.75, 1.32)	0.936
25-34 years	3,846	483 (12.6)	0.85 (0.68, 1.05)	0.046	124.0 (36.5, 308.5)	1.04 (0.89, 1.22)	0.527
35-44 years	4,492	722 (16.1)	0.98 (0.81, 1.19)	0.807	134.0 (30.2, 324.0)	1.08 (0.94, 1.24)	0.159
45-54 years	4,724	720 (15.2)	1.00 (ref)	-	126.0 (41.8, 326.2)	1.00 (ref)	-
55-64 years	2,878	380 (13.2)	0.84 (0.67, 1.04)	0.039	103.0 (22.8, 287.2)	1.07 (0.90, 1.27)	0.3
65 or more years	235	28 (11.9)	0.92 (0.46, 1.76)	0.752	135.0 (35.5, 314.5)	0.82 (0.49, 1.36)	0.317
Employment type							
Full time employee	12,137	1,746 (14.4)	1.00 (ref)	-	118.0 (33.0, 307.8)	1.00 (ref)	-
Part time employee	3,071	388 (12.6)	1.00 (0.79, 1.26)	0.994	137.0 (36.5, 334.0)	0.88 (0.74, 1.04)	0.049
Others	2,481	297 (12.0)	0.93 (0.74, 1.17)	0.433	151.0 (38.0, 334.0)	0.97 (0.82, 1.15)	0.663
Employer size				-			
Large	5,484	752 (13.7)	1.08 (0.90, 1.29)	0.286	118.0 (34.8, 288.2)	1.06 (0.92, 1.22)	0.279
Medium	7,161	978 (13.7)	1.00 (ref)	-	139.5 (40.0, 338.0)	1.00 (ref)	-
Small	4,137	632 (15.3)	1.17 (0.97, 1.41)	0.028	111.0 (23.0, 312.2)	1.04 (0.91, 1.19)	0.398
Missing	907	69 (7.6)		-	71.0 (32.0, 246.0)		

Occupation				-			
Associate Professionals	1,778	204 (11.5)	0.97 (0.69, 1.34)	0.804	108.0 (24.0, 327.0)	1.09 (0.85, 1.40)	0.365
Elementary clerical, sales and service workers	714	109 (15.3)	1.31 (0.88, 1.92)	0.075	136.0 (39.0, 337.0)	1.14 (0.86, 1.51)	0.231
Intermediate clerical, sales and service workers	2,419	290 (12.0)	0.85 (0.64, 1.11)	0.116	115.5 (37.2, 275.8)	1.08 (0.88, 1.32)	0.335
Intermediate production and transport workers	3,476	530 (15.2)	1.07 (0.85, 1.33)	0.469	141.5 (41.2, 323.8)	1.00 (0.84, 1.17)	0.942
Labourers and related workers	3,668	515 (14.0)	1.00 (ref)	-	146.0 (45.0, 341.0)	1.00 (ref)	-
Managers and administrators	501	90 (18.0)	1.15 (0.76, 1.73)	0.384	92.0 (33.0, 213.5)	1.33 (0.98, 1.80)	0.015
Professionals	2,008	269 (13.4)	1.10 (0.82, 1.47)	0.418	111.0 (28.0, 279.0)	1.16 (0.94, 1.45)	0.073
Tradespersons and related workers	3,125	424 (13.6)	1.09 (0.86, 1.38)	0.371	106.5 (19.5, 308.8)	1.11 (0.93, 1.33)	0.119
Socioeconomic status				-			
Fifth quintile	3,046	327 (10.7)	0.88 (0.71, 1.09)	0.129	116.0 (30.0, 260.0)	1.13 (0.96, 1.34)	0.053
Middle three quintiles	11,742	1,634 (13.9)	1.00 (ref)	-	119.0 (33.0, 324.0)	1.00 (ref)	-
First quintile	2,868	462 (16.1)	0.98 (0.81, 1.19)	0.838	144.0 (44.0, 336.8)	0.92 (0.80, 1.06)	0.147
Missing	33	8 (24.2)		-	57.0 (25.0, 109.0)		
Remoteness				-			
Major Cities	12,830	1,719 (13.4)	1.00 (ref)	-	120.0 (36.0, 300.0)	1.00 (ref)	-
Inner Regional	4,022	604 (15.0)	1.14 (0.95, 1.35)	0.063	140.5 (34.8, 358.2)	1.01 (0.89, 1.15)	0.82
Outer Regional and Remote	818	103 (12.6)	0.82 (0.57, 1.17)	0.152	104.0 (7.5, 307.5)	1.27 (0.96, 1.67)	0.026
Missing	19	5 (26.3)		-	44.0 (33.0, 107.0)		
Dispensed gabapentin in same 2-year period							
Dispensed gabapentin	395	218 (55.2)	1.40 (1.02, 1.92)*	0.006	136.0 (40.8, 316.2)	1.02 (0.84, 1.23)	0.793
No gabapentin	17,294	2,213 (12.8)	1.00 (ref)	-	120.0 (34.0, 319.0)	1.00 (ref)	-
Dispensed opioid analgesics in same 2-year period				-			
Dispensed opioid analgesics	5,541	2,206 (39.8)	19.55 (15.90, 24.20)*	p<0.001	124.0 (34.0, 320.0)	1.11 (0.92, 1.35)	0.15
No opioids	12,148	225 (1.9)	1.00 (ref)	-	123.0 (35.0, 295.0)	1.00 (ref)	-
Dispensed antidepressants in same 2-year period				-			
Dispensed antidepressants	2,476	1,347 (54.4)	3.61 (3.06, 4.27)*	p<0.001	145.0 (49.0, 334.5)	0.89 (0.79, 0.99)*	0.007
No antidepressants	15,213	1,084 (7.1)	1.00 (ref)	-	95.0 (21.0, 289.5)	1.00 (ref)	-

Dispensed benzodiazepines in same 2-year period				-			
Dispensed benzodiazepines	2,176	1,026 (47.2)	1.51 (1.27, 1.79)*	p<0.001	143.0 (42.0, 348.5)	0.96 (0.85, 1.08)	0.349
No benzodiazepines	15,513	1,405 (9.1)	1.00 (ref)	-	117.0 (31.0, 294.0)	1.00 (ref)	-
